# A gel aging effect in the synthesis of open-framework gallium phosphates: structure solution and solid-state NMR of a large-pore, open-framework material†
†Electronic supplementary information (ESI) available. CCDC 1577782 and 1577783. For ESI and crystallographic data in CIF or other electronic format see DOI: 10.1039/c7dt03709k


**DOI:** 10.1039/c7dt03709k

**Published:** 2017-11-16

**Authors:** Lucy K. Broom, Guy J. Clarkson, Nathalie Guillou, Joseph E. Hooper, Daniel M. Dawson, Chiu C. Tang, Sharon E. Ashbrook, Richard I. Walton

**Affiliations:** a Department of Chemistry , University of Warwick , Coventry , CV4 7AL , UK . Email: r.i.walton@warwick.ac.uk; b Institut Lavoisier Versailles , UMR CNRS 8180 , Université de Versailles St-Quentin-en-Yvelines , Université Paris-Saclay , 78035 Versailles , France; c School of Chemistry , EaStCHEM and Centre of Magnetic Resonance , University of St Andrews , North Haugh , St Andrews , KY16 9ST , UK; d Diamond Light Source , Diamond House , Harwell Science and Innovation Campus , Fermi Ave , Didcot OX11 0DE , UK

## Abstract

The templated zeolite-analogue GaPO-34 (CHA structure type) crystallises from a gel precursor Ga_2_O_3_ : 2H_3_PO_4_ : 1HF : 1.7SDA : 70H_2_O (where SDA = structure directing agent), treated hydrothermally for 24 hours at 170 °C using either pyridine or 1-methylimizadole as SDA and one of either poorly crystalline ε-Ga_2_O_3_ or γ-Ga_2_O_3_ as gallium precursor. If the same gels are stirred for periods shorter than 2 hours but treated under identical hydrothermal conditions, then a second phase crystallises, free of GaPO-34. If β-Ga_2_O_3_ is used as a reagent only the second phase is found to crystallise, irrespective of gel aging time. The competing phase, which we denote GaPO-34A, has been structurally characterised using synchrotron powder X-ray diffraction for the pyridine material, GaPO-34A(pyr), and using single-crystal X-ray diffraction for the 1-methylimiazole material, GaPO-34A(mim). The structure of GaPO-34A(pyr), *P*1, *a* = 10.22682(6) Å, *b* = 12.09585(7) Å, *c* = 13.86713(8) Å, *α* = 104.6531(4)°, *β* = 100.8111(6)°, *γ* = 102.5228(6)°, contains 7 unique gallium sites and 6 phosphorus sites, with empirical formula [Ga_7_P_6_O_24_(OH)_2_F_3_(H_2_O)_2_]·2(C_5_NH_6_). GaPO-34A(mim) is isostructural but is modelled as a half volume unit cell, *P*1, *a* = 5.0991(2) Å, *b* = 12.0631(6) Å, *c* = 13.8405(9) Å, *α* = 104.626(5)°, *β* = 100.346(5)°, *γ* = 101.936(4)°, with a gallium and a bridging fluoride partially occupied and two partially occupied SDA sites. Solid-state ^31^P and ^71^Ga NMR spectroscopy confirms the structural complexity of GaPO-34A with signals resulting from overlapping lineshapes from multiple Ga and P sites, while ^1^H and ^13^C solid-state NMR spectra confirm the presence of the protonated SDA and provide evidence for disorder in the SDA. The protonated SDA is located in 14-ring one-dimensional channels with hydrogen bonding deduced from the SDA nitrogens to framework oxygen distances. Upon thermal treatment to investigate SDA removal, structure collapse occurs, which may be due the large number of bridging hydroxides and fluorides in the as-made material, and the unequal amounts of gallium and phosphorus present.

## Abstract 2

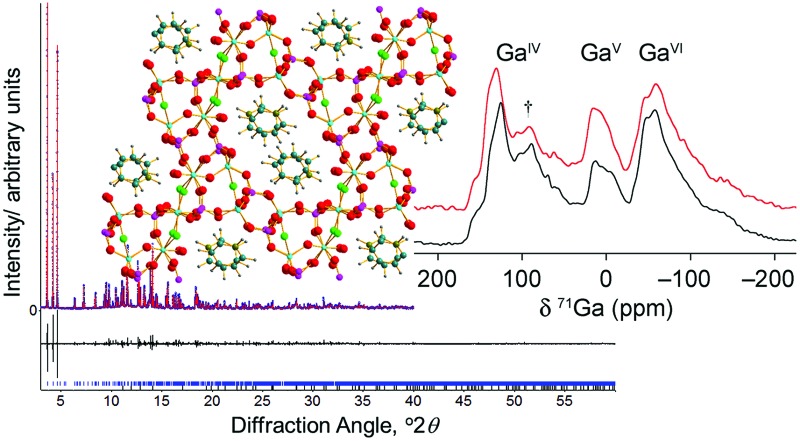
History of gel precursors affects the resulting product in gallium phosphate crystallisation allowing isolation of a 14-ring channel structure.

## Introduction

Gallium phosphate (GaPO) ‘zeotypes’ have been studied for a number of years and, while some have structures related to those of silicate zeolites and others are analogues of the large family of aluminium phosphates (AlPOs), they present several unique open framework structures not (yet) found for other compositions.^1^ As with aluminium in AlPOs, gallium in GaPOs may be found in either four-, five- or six-coordinate environments, but in as-made GaPOs the gallium coordination is always completed by hydroxide or fluoride ions in five- and six-coordinate sites and gallium is never found to be solely four-coordinate.^2^ A great variety in the gallium phosphate family was introduced by the synthesis of fluorinated versions of these materials, such as in the ULM-n^3^ and Mu-n^4^ families; these have been studied as a model system for understanding both the hydrothermal synthesis of open-framework solids using *in situ* crystallisation probes,^5^ and the thermodynamic stability of their structures using computational methods.^6^,^7^

In the synthesis of open-framework materials it is well established that from a given set of reagents a number of materials may crystallise depending on experimental factors such as reagent ratios, synthesis time and reaction temperature.^8^ For example, the formation of increasingly dense framework structures with increased reaction time or temperature follows Ostwald's rule, where the successive transformation of phases into increasingly thermodynamically stable materials occurs.^8^ It can be necessary, therefore, to explore widely the possible reaction conditions in order to discover new materials that may have porosity suitable for practical applications. The phenomenon of gel aging is known in silicate zeolite chemistry where the crystallisation kinetics are enhanced and the crystallinity of a zeolite is improved after allowing the precursor gel to stand for a period of time before heating to the reaction (crystallisation) temperature.^9^ In some cases, the crystalline product formed depends on the time of aging of the gel, for example the formation of zeolite X from a gel used to prepare zeolite P can be induced by pre-aging the gel at room temperature before hydrothermal treatment.^9^ In other cases the aging time can significantly change the phase outcome of reactions, for example, in the production of FAU/LTA zeolites no aging time allows for the production of almost pure LTA, whereas more FAU is formed at longer aging times.^10^ It has been speculated by Ogura *et al.*, that less aging is preferred for developing structures composed of mainly four-ringed structures, such as SOD.^11^ It has also been postulated that structures composed of mainly six-ring building units (including CHA, ANA and FAU) arise from longer periods of ageing.^10^

Here we report a new example of a gel aging effect, in which the preparation of one gallium phosphate framework over another depends on the time of aging of the gel precursor. The choice of gallium oxide reagent also affects the outcome of crystallisation, implying its important role in the gel chemistry. These observations arose from a reinvestigation of the work of Schott-Darie *et al.* who reported two GaPOs in the Ga_2_O_3_-P_2_O_5_-SDA-HF-H_2_O (structure-directing agent (SDA) = pyridine (pyr) or 1-methylimizadole (mim)) system: CHA-type GaPO-34 and an unidentified phase, and proposed that the water content of the gel led to the formation of one phase over the other.^12^ We demonstrate that, in fact, the aging time of the gel is the key factor in determining which phase forms. This information allowed for the preparation of samples of the unknown GaPO containing both SDAs for structure solution and characterisation of their thermal decomposition. The structure of the 1-methylimidazole form, GaPO-34A(mim), was solved from a small single crystals, while that of the pyridine form, GaPO-34A(pyr), was solved from high-resolution synchrotron powder X-ray diffraction. Although it has previously been found that different GaPOs may be formed from the same gel precursor at room temperature compared to with heating,^13^ we believe that the example reported herein is the first example of a gel aging effect in phosphate chemistry leading to distinct products upon subsequent hydrothermal treatment. Previously, only changes in morphology and crystal size have been reported in AlPOs with aging of the precursor gel.^14^

## Experimental section

Synthesis from gels of composition 1Ga_2_O_3_ : 2H_3_PO_4_ : 1HF : *n*H_2_O : 1.7SDA was studied (SDA = pyridine (pyr) or 1-methylimidazole (mim)) as originally used by Schott-Darie *et al.* for the preparation of GaPO-34.^12^ Three different Ga_2_O_3_ precursors were investigated: (i) nanocrystalline ε-Ga_2_O_3_, a poorly crystalline material prepared by the thermal decomposition of Ga(NO_3_)_3_·9H_2_O.^15^ The method of Birnara *et al.* was used to prepare Ga(NO_3_)_3_·9H_2_O, by dissolving 5.6 g of gallium metal (99.9%, Sigma-Aldrich) in 50 mL concentrated nitric acid (70%, laboratory reagent grade, Fisher Scientific) by stirring for 24 hours.^16^ The colourless solution was then chilled to 5 °C and a crystal of Ga(NO_3_)_3_·9H_2_O was used to seed crystallisation. The crystals formed were filtered and dried for several days at room temperature and then heated in an oven at 240 °C for 24 hours to give ε-Ga_2_O_3_; (ii) γ-Ga_2_O_3_, prepared using the method of Playford *et al.* from 8 mL of ethanolamine (99%, Merck) mixed with 0.3 g of gallium metal, which was placed in a 25 mL Teflon-lined autoclave and heated to 240 °C for 72 hours.^15^ The solid product was then filtered and washed with methanol, before drying in an oven at 70 °C; (iii) β-Ga_2_O_3_, used as supplied by Sigma-Aldrich (99.99+%) after confirmation of its purity by powder X-ray diffraction. To prepare the precursor gel, the gallium oxide was dispersed in deionised water in a ∼25 ml Teflon™ autoclave liner with stirring before addition of HF (40% in water Fluka), H_3_PO_4_ (85% in water Fisher Chemicals) and, finally, the SDA. In all reactions 0.25 g of Ga_2_O_3_ was used, while the amount of water was varied between *n* = 50 and 70 molar equivalents. The gel was stirred at room temperature for a chosen period of time before being sealed in the autoclave, transferred to a pre-heated fan oven and held at 170 °C for 24 hours. The autoclaves were allowed to cool naturally to room temperature before the solid product was recovered by vacuum filtration, washed with copious amounts of deionised water and dried at 70 °C in air overnight before further study. Teflon™ liners were cleaned between reactions by heating with concentrated (∼8 M) NaOH at 170 °C to avoid contamination and seeding effects, and selected reactions were repeated to ensure reproducibility of results.

Powder X-ray diffraction (PXRD) was used to identify the solid products with data recorded using a Siemens D5000 diffractometer operating with Cu K_α1/2_ radiation in flat-plate geometry. Thermogravimetric analysis (TGA) and differential scanning calorimetry (DSC) were performed using a Mettler Toledo TGA/DSC 1-600 instrument under static air with a heating rate of 10 °C min^–1^ from room temperature to 1000 °C. In further TGA-DSC experiments, simultaneous mass spectra were recorded using a Hiden HPR-20 QIC R&D specialist gas analysis system, a triple-filter mass spectrometer with SEM detection on heating in nitrogen to 900 °C at 10 °C min^–1^, with nitrogen as carrier gas to minimise background water and to eliminate CO_2_ from the background. Thermodiffraction experiments were carried out using a Bruker D8 powder X-ray diffractometer operating with Cu K_α1/2_ radiation and fitted with a HTK900 gas chamber and VÅNTEC-1 detector. Patterns were recorded on heating from room temperature to 900 °C in intervals of 10 °C with a 10 minutes equilibration time before scans lasting 10 minutes were made. Infrared spectra were recorded using a PerkinElmer 100 FTIR spectrometer with a Diamond ATR stage. Elemental analysis was performed by Medac Ltd for Ga and P using ICP-OES after digestion and for F using Schöniger flask oxygen combustion followed by titration.

Single-crystal X-ray diffraction data were recorded using the UK national crystallography service.^17^ A suitable crystal of GaPO-34A(mim) was selected and mounted on a Mitegen head and placed on a Rigaku FRE+ equipped with VHF Varimax confocal mirrors and an AFC10 goniometer with an HG Saturn 724+ detector. The crystal was kept at 100(2) K during data collection. Using Olex2,^18^ the structure was solved with the ShelXS^19^ structure solution program using Direct Methods and refined with the ShelXL^20^ refinement package using least-squares minimisation.

High-resolution PXRD data were recorded from a sample of GaPO-34A(pyr) at room temperature using Beamline I11 of the Diamond Light Source.^21^ The material was contained in a thin-wall silica capillary and data recorded using a wavelength of 0.82640(1) Å with a wide-angle position-sensitive detector based on Mythen-2 Si strip modules, which allows fast data collection (a few seconds per pattern) to avoid beam damage.^22^ All calculations of the structural investigation of GaPO-34A(pyr) were performed with the TOPAS program.^23^ The LSI-indexing method based on the 20 first peaks common to two different patterns measured from two different samples converged first to a triclinic unit cell similar to that of GaPO-34A(mim) (*a* = 5.1164 Å, *b* = 12.1058 Å, *c* = 13.8724 Å, *α* = 104.6750°, *β* = 100.8040° *γ* = 102.5350°, and *V* = 784.2 Å^3^) with a satisfactory figure-of-merit (*M*_20_ = 145). First attempts to solve the structure from powder diffraction data in this unit cell did not converge to a satisfactory model, however, taking into the account similarity between this unit cell and that of the structural models of the disordered GaPO-34A(mim) solved from single crystal data, it was assumed that GaPO-34A(pyr) was also isostructural to DIPYR-GaPO^24^ (as described below), even if superstructure peaks of the double unit cell were not evidenced. Therefore, atomic coordinates of DIPYR-GaPO were directly used as starting model in the Rietveld refinement and the direct space strategy was then used to localise pyridinium cations, which were added and treated as rigid bodies in a simulated annealing process. A careful examination of N–H···O and C–H···O contacts allowed two orientations to be chosen for the pyridinium, but it is clear that more extensive disorder of the SDAs is likely to be present, which cannot be resolved by diffraction alone. OH and F anions were chosen to be in the same framework positions as in the isostructural DIPYR-GaPO^24^ (see below). The anisotropic line broadening effect was modelled using a spherical harmonics series. At the final stage, Rietveld refinement involved the following structural parameters: 132 atomic coordinates, 12 parameters for the localization of the organic molecules, 1 mean C–C, C–N distance, 4 thermal factors and 1 scale factor for 5951 reflections. The final Rietveld model gave a satisfactory model indicator (*R*_B_ = 0.036) and profile factors (*R*_p_ = 0.088 and *R*_wp_ = 0.116). The structural model of the quartz-type GaPO_4_ (berlinite),^25^ identified as an impurity, was taken into account, and its quantitative amount was estimated to be about 5 wt%.

Solid-state NMR spectra were recorded on Bruker Avance III spectrometers equipped with 9.4, 14.1 or 20.0 T wide-bore superconducting magnets at the University of St Andrews (9.4 and 14.1 T) or the UK 850 MHz Solid-State NMR facility at the University of Warwick (20.0 T). For ^1^H NMR spectra, samples were packed into 1.3 mm ZrO_2_ rotors and rotated at the magic angle at 50 or 55 kHz. To minimize signal from the probe background, spectra were recorded using a rotor-synchronized spin-echo pulse sequence with an echo delay of one rotor period (18.2 or 20.0 μs). Signal averaging was carried out for 128 transients with a recycle interval of 3 s. ^1^H double-quantum (DQ) correlation spectra were recorded using two blocks of rotor-synchronised back to back (BABA) pulses to excite and convert DQ coherences.^26^ Signal averaging was carried out for 16 or 32 transients for each of 148 *t*_1_ increments of 16.6 μs. For ^13^C NMR spectra, samples were packed into 4 mm ZrO_2_ rotors and rotated at the magic angle at a rate of 12.5 kHz. Spectra were recorded using cross-polarization (CP) from ^1^H with a spin lock pulse (ramped for ^1^H) between 0.5 and 2.5 ms. TPPM-15 decoupling of ^1^H (*ν*_1_ ≈ 100 kHz) was applied during acquisition. Signal averaging was carried out for between 2048 and 5120 transients with a recycle interval of 3 s. For ^31^P NMR spectra, samples were packed into 4 mm rotors and rotated at the magic angle at a rate of 14 kHz. Spectra were recorded with signal averaging for 8 transients with a recycle interval of 60 s. To eliminate the signal from the GaPO_4_ berlinite impurity, additional spectra were recorded with CP from ^1^H with a spin-lock pulse (ramped for ^1^H) of 1 ms. Signal averaging was carried out for 256 transients with a recycle interval of 3 s. SPINAL-64 decoupling of ^1^H (*ν*_1_ ≈ 100 kHz) was applied during acquisition. For ^71^Ga NMR spectra, samples were packed into 1.3 mm rotors and rotated at the magic angle at 55 kHz. To minimize distortion of the spectral lineshapes, spectra were recorded using a rotor-synchronized spin-echo pulse sequence with an echo delay of one rotor period (18.2 μs). Signal averaging was carried out for 8192 or 16 384 transients with a recycle interval of 0.5 s. Chemical shifts are reported in ppm relative to TMS for ^1^H and ^13^C, 85% H_3_PO_4 (aq)_ for ^31^P and 1 M Ga(NO_3_)_3 (aq)_ using secondary reference compounds of l-alanine for ^1^H and ^13^C (^1^H: N**H***δ* = 8.5 ppm, ^13^C **C**H_3_*δ* = 20.5 ppm), for ^31^P BPO_4_ (*δ* = –29.6 ppm) and for ^71^Ga GaPO_4_ berlinite (*δ*_iso_ = –111.1 ppm, *C*_Q_ = 8.8 MHz, *η*_Q_ = 0.45).

## Results and discussion

**Fig. 1:**
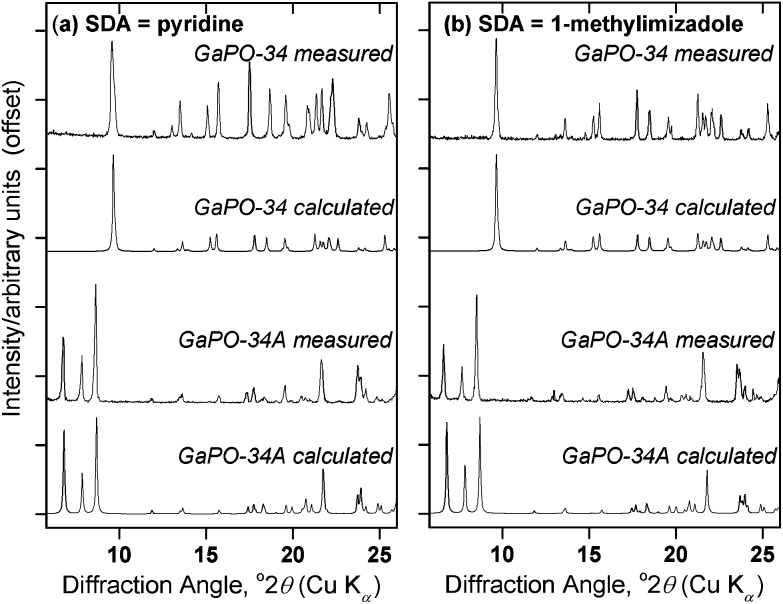
PXRD patterns of materials prepared using (a) pyridine and (b) 1-methylimidazole as the structure directing agent, from gels of composition 1Ga_2_O_3_ : 2H_3_PO_4_ : 1HF : 70H_2_O : 1.7SDA heated at 170 °C. For each SDA, GaPO-34A was prepared with β-Ga_2_O_3_ as the gallium source and 1 hour gel aging before heating for 87 hours, while for GaPO-34, ε-Ga_2_O_3_ was used and the gel was aged for 3 hours before being heated for 24 hours. The calculated patterns for GaPO-34A are from the crystal structures described below, while those for GaPO-34 are from the 1-methylimizadole form of the material (in the absence of a structure for the pyridine form) as reported by Schott-Darie *et al.*^12^

Our synthetic approach was based on that of Schott-Darie *et al.* using a gel precursor prepared from gallium oxide, orthophosphoric acid, HF, the desired SDA and water.^12^ We previously used this method to prepare GaPO-34 27 and in that case the reagent mixture had been stirred for a period in excess of 5 hours to ensure gel homogeneity before hydrothermal treatment at 170 °C for 24 hours. During attempts to replicate this synthesis, it became apparent that shorter periods of stirring prior to the same hydrothermal treatment resulted in the formation of the unidentified GaPO, with a PXRD pattern resembling that reported by Schott-Darie *et al.*,^12^Fig. 1. Schott-Darie *et al.* named this phase GaPO-A, but herein we use the name GaPO-34A, to denote the fact that the material crystallises from the same gel as GaPO-34. This nomenclature is consistent with synthetically related materials: for example AlPO-14A crystallises as a competing phase from a synthesis of AlPO-14 yet has a distinctly different structure.^28^

Table 1 provides details of exploratory syntheses with pyridine as structure-directing agent aimed at identifying reaction conditions for isolating GaPO-34A over GaPO-34. Periods of aging the gel of 2 or more hours always yields GaPO-34, when either poorly crystalline ε-Ga_2_O_3_ or γ-Ga_2_O_3_ are used as gallium sources, while aging for 1 hour gives GaPO-34A (contaminated with a small amount of the dense GaPO_4_, berlinite). Interestingly, when β-Ga_2_O_3_ is used as the gallium source, only GaPO-34A is formed after hydrothermal treatment, even after extended aging time (>6 days) before heating. One difference between this precursor and the other two is its highly crystalline nature (see Fig. S1 and S2†), which may mean that dissolution and reaction under hydrothermal conditions affects the nucleation and kinetics of crystal growth of the gallium phosphate products. The same is true whether pyridine or 1-methylimizadole is used as the structure directing agent (see Table S1, ESI†). In further synthesis experiments with β-Ga_2_O_3_ as the gallium source, we extended the crystallisation time to 87 hours after a 1 hour gel aging but this still yielded only GaPO-34A (not shown in Table 1). Schott-Darie *et al.* suggested that the water content of the gel may dictate whether GaPO-34A or GaPO-34 is formed but, as shown in Table 1, reducing the water content from 70 to 50 molar equivalents has no effect on the outcome of reaction.

**Table 1:** Results of exploratory syntheses of gallium phosphates from gels of composition 1Ga_2_O_3_ : 2H_3_PO_4_ : 1HF : *n*H_2_O : 1.7pyr. After aging all were heated at 170 °C for 24 hours

Ga_2_O_3_ source	Water equivalents (*n*)	Aging time/hours	GaPO product from PXRD
Poorly crystalline ε-Ga_2_O_3_	70	0	GaPO-34A
	70	1	GaPO-34A
	60	1	GaPO-34A
	50	1	GaPO-34A
	70	2	GaPO-34
	70	3	GaPO-34
	70	4	GaPO-34
	50	3	GaPO-34
	60	3	GaPO-34
γ-Ga_2_O_3_	70	1	GaPO-34A
	70	2	GaPO-34
	70	3	GaPO-34
	70	4	GaPO-34
β-Ga_2_O_3_	70	1	GaPO-34A
	70	2	GaPO-34A
	70	3	GaPO-34A
	70	18	GaPO-34A
	70	24	GaPO-34A
	70	146	GaPO-34A

IR spectra of GaPO-34A, Fig. S3, ESI,† show the presence of the expected phosphate vibrations (1250–750 cm^–1^) and provide evidence for Ga–F bonds, at ∼520 cm^–1^,^29^ as well as the presence of the organic SDA in each sample by the presence of bands in the fingerprint region (1500 to 500 cm^–1^). There is some evidence for the occlusion of water in the GaPO-34A materials from the features in the region of the O–H stretch at ∼3250 cm^–1^, and this is consistent with the structure determination described below, although note that the framework also contains hydroxide anions that will show IR features with similar energies.

**Fig. 2:**
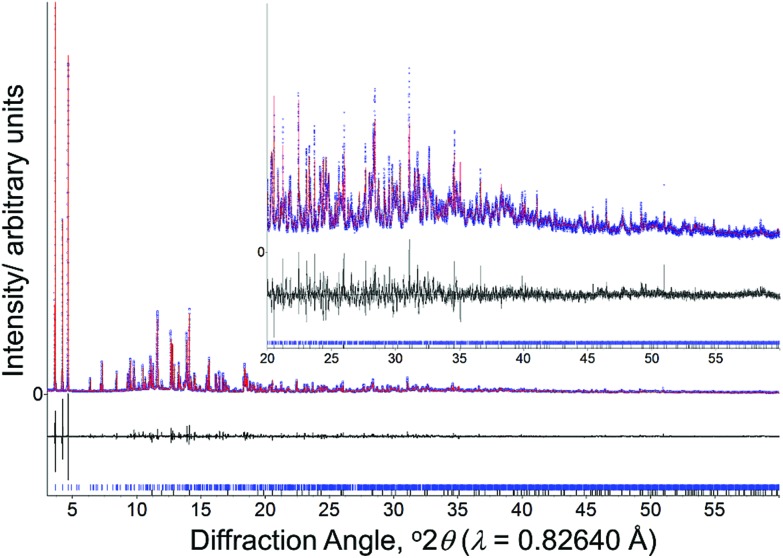
Final Rietveld plot for GaPO-34A(pyr). Blue points are the experimental synchrotron X-ray data, the red line is the fitted profile, the black line is the difference and the blue ticks are the allowed Bragg peak positions.

Having prepared highly crystalline samples of GaPO-34A, structure solution was carried out. In the case of GaPO-34A(mim), a small single crystal was selected and sufficient data recorded for structure solution and refinement. For GaPO-34A(pyr), structure solution from high-resolution PXRD was required as no suitable single crystals could be found: here a sample prepared with 1 hour gel aging and a crystallisation time of 87 hours was studied. Fig. 2 shows the final Rietveld plot for the pyridine material, while Table 2 summarises the crystal data from each material.

**Table 2:** Crystal data for two forms of GaPO-34A

	GaPO-34A(pyr)	GaPO-34A(mim)
Empirical formula	Ga_7_P_6_F_3_O_28_C_10_ H_18_N_2_	Ga_7_P_6_F_2.67_O_28_C_6.66_H_9.99_N_3.33_
*M*_r_/g mol^–1^	1345.1	1309.3
Crystal system	Triclinic	Triclinic
Space group	*P*1	*P*1
*a*/Å	10.22682(6)	5.0991(2)
*b*/Å	12.09585(7)	12.0631(6)
*c*/Å	13.86713(8)	13.8405(9)
*α*/°	104.6531(4)	104.626(5)
*β*/°	100.8111(6)	100.346(5)
*γ*/°	102.5228(6)	101.936(4)
*V*/Å^3^	1565.84(2)	781.41(8)
*Z*	2	1
Data collection	Powder	Single-crystal
*λ*/Å	0.825781	0.709300
Temperature/K	298	100
Number of reflections	5951	3585
Number of fitted structural parameters	150	242
Number of restraints	60	76
Goodness of fit parameters	*R*_p_ = 0.088	*R*_int_ = 0.0703
	*R*_wp_ = 0.116	*R*_*σ*_ = 0.0737
	*R*_Bragg_ = 0.036	*R*_1_ = 0.0607 (*I* > 2*σ*(*I*))
	GoF = 3.03	w*R*_2_ = 0.1472 (all data)

**Fig. 3:**
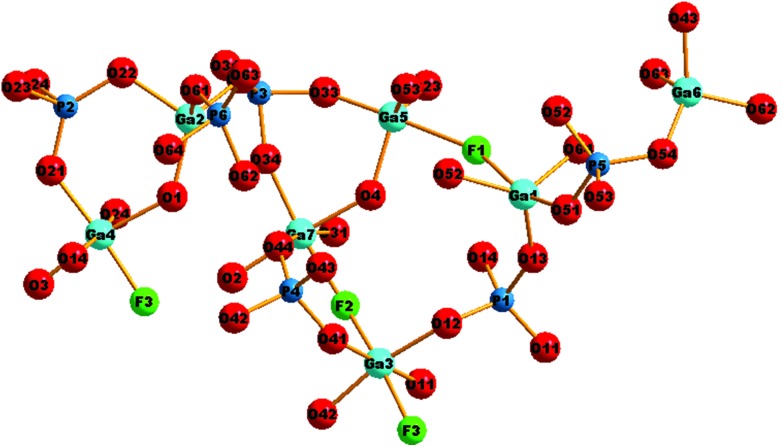
The local environment and connectivity of the crystallographically unique Ga and P sites in GaPO-34A(pyr).

The structure of GaPO-34A (with either SDA) is a new variant of ‘DIPYR-GaPO’ reported by Weigel *et al.*^24^ In the earlier work the material was prepared hydrothermally using two organic additives, pyridine and benzylviologen dichloride, where the latter decomposed during the reaction to form 4,4′-dipyridyl, but only a single crystal was selected from a mixture of products: the sample was not phase pure. Here, we will use GaPO-34A(pyr) to describe the structure of the material before comparing with GaPO-34A(mim) and DIPYR-GaPO. GaPO-34A(pyr) has composition [Ga_7_P_6_O_24_(OH)_2_F_3_(H_2_O)_2_]·2(C_5_NH_6_) and consists of an anionic gallium fluorohydroxyphosphate framework with occluded pyridinium cations along with water molecules that are directly coordinated to some of the framework galliums. The unit cell contains seven crystallographically distinct sites for gallium and 6 for phosphorus, Fig. 3.

**Fig. 4:**
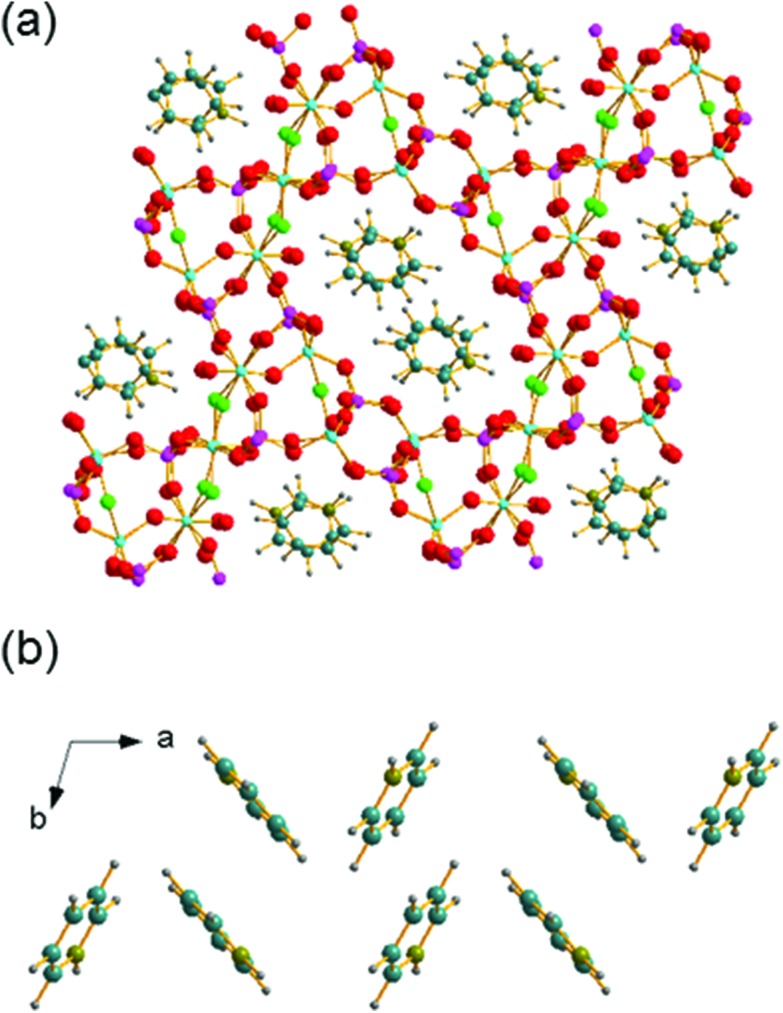
Views of the crystal structure of GaPO-34A(pyr) (a) viewed along the *a* axis and (b) the stacking of the pyridinium cations. The pale blue spheres represent gallium, pink phosphorus, red oxygen, bright green fluoride, teal carbon, grey hydrogen and olive nitrogen.

Each of the phosphorus sites is tetrahedrally coordinated by four oxygens, with P–O distances in the expected range from 1.49(1) Å (P6–O64) to 1.589(8) Å (P1–O14), each of which in turn bridges to a gallium. In contrast, the gallium sites have a variety of coordination numbers, geometries and coordinated atoms. Two of the galliums are four-coordinate (approximately tetrahedral), with one of these (Ga6) having four Ga–O–P linkages and the other (Ga2) having three Ga–O–P linkages and one hydroxide that bridges to Ga4. Two of the galliums are five-coordinate: Ga1 has four oxygens shared with phosphorus and also has a fluoride that bridges to Ga5, and in turn Ga5 has three Ga–O–P linkages and a hydroxide ion that bridges to Ga7. The remaining three galliums are six-coordinate: Ga3 has four Ga–O–P linkages and two fluorides that bridge to Ga4 and Ga7, while, in turn, Ga4 and Ga7 each have three Ga–O–P linkages, one fluoride bridging to Ga3, one hydroxide bridging to another gallium and a terminal water molecule. Bond valence sums confirm that each Ga is trivalent and phosphorus is pentavalent, with bond distances as expected in related gallium phosphate materials (see ESI†). The various primary building units are linked to give an open-framework structure that contains one-dimensional channels bounded by 14-rings of Ga and P centres running parallel to the *a* axis, as seen in Fig. 4a. The coordinated water molecules sit in the channels, which are also occupied by the charge-balancing pyridinium cations, which are stacked in two columns: the neighbouring pyridinium cations do not, however, lie face-to-face, rather they form a herring-bone-like pattern, Fig. 4b. There is evidence for hydrogen-bonding interactions between the pyridinium cations and the inorganic framework: N–O distances of 2.971 Å (N21–O51) and 2.982 (N1–O12) are observed.

The structure of GaPO-34A(mim) is essentially the same as GaPO-34A(pyr), but the structure was refined from single-crystal XRD with a unit cell of close to half the volume, *i.e.* close to half the value for the *a* axis: see Table 2, and Tables S2 and S4,† for comparison of bond distances between the two structures. The consequence of the half unit cell volume is that some fractional occupancy of certain atom sites is found. Consequently, while there are only four crystallographically-unique gallium sites, one of these is partially occupied (67%) with a close, alternative site (33% occupancy), and two of these are connected by a partially-occupied bridging fluoride, thus leading to seven possible individual types of gallium. There are three crystallographic phosphorus sites but the disorder of the gallium environments, the partially occupied fluoride and the fact that the 1-methylimizadolium SDA is modelled as having two orientations, may generate local static disorder (see the solid-state NMR discussion below). To ensure charge balance the occupancy of the bridging fluoride was coupled to the total occupancy of one orientation of the SDA. It may equally be possible that some unprotonated SDA is present in the structure, but because of the small, weakly diffracting crystal of this sample, we were unable to refine the structure to any greater level of detail from the data measured; similarly the data provide no evidence for the larger volume unit cell found for the pyridine analogue. The overall composition of GaPO-34A(mim) is, thus, modelled from the single crystal analysis as [Ga_7_P_6_O_24_(OH)_2_(H_2_O)_2_F_2.67_](C_4_N_2_H_7_)_1.67_, with the anionic charge of the framework balanced by 1-methylimizadolium cations. Elemental analysis for Ga, P and F gave molar ratios of 1.00 : 0.93 : 0.26 for GaPO-34A(mim) (expected from the single crystal structure 1.00 : 0.85 : 0.38) and 1.00 : 0.88 : 0.32 for GaPO-34A(pyr) (expected from the powder diffraction structure 1.00 : 0.85 : 0.43). Analysis for CHN by combustion was inconclusive, and as will be explained below this is due to retention of carbon in the solid after thermal collapse.

**Fig. 5:**
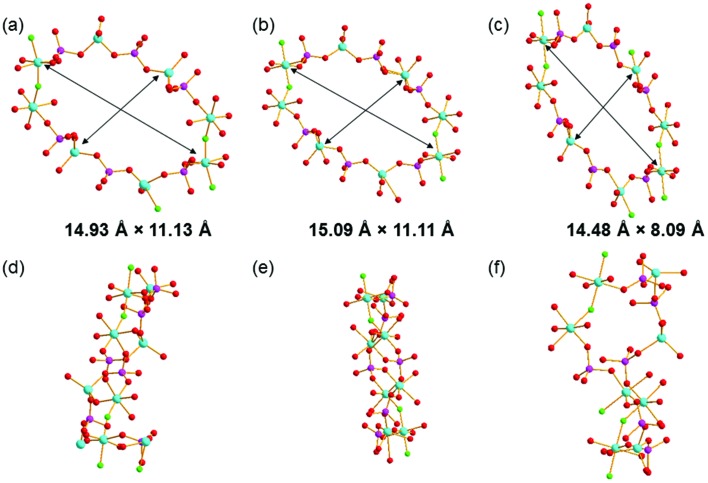
The 14-ring channels in (a) and (d) GaPO-34A(pyr), (b) and (e) in GaPO-34A(mim), and (c) and (f) in DIPYR-GaPO. In (a)–(c), the structure is viewed along the *a*-axis and in (d)–(f) along the *c*-axis and the atom colouring scheme is the same as in Fig. 3. In (a)–(c), the distances indicated are measured between equivalent Ga centres in each structure: Ga3–Ga3 and Ga2–Ga2 in GaPO-34A(pyr), Ga4–Ga4 and Ga2–Ga2 in GaPO-34A(mim) and Ga3–Ga3 and Ga2–Ga5 in DIPYR-GaPO.

It can be noted that the structure of GaPO-34A bears no resemblance to that of as-made GaPO-34 (prepared using the same SDAs): for GaPO-34 the inorganic framework is that of the chabazite structure consisting of a stacked sequence of 6-rings and, in the as-made form, contains a pair of bridging fluorides that create a six-coordinate gallium site in addition to two tetrahedral sites.^27^ In addition, the Ga : P ratio is 1 : 1 in GAPO-34, in contrast to 7 : 6 in GaPO-34A. Comparing the structure of GaPO-34A and the previously published DIPYR-GaPO^24^ shows that the materials have the same topologies but the conformation of the inorganic framework differs significantly between the two, most clearly seen by examining the geometry of the 14-rings that make up the 1D channels, as shown in Fig. 5. Differences can be seen between the two forms of GaPO-34A with slightly more elliptical channels present when the SDA is 1-methylimizadole. However, the channels of DIPYR-GaPO are much more compressed in both directions and, when viewed in a perpendicular direction, the twisted nature of the 14-rings can be observed, compared to the planar orientation in the two forms of GaPO-34A. The structure of DIPYR-GaPO was determined from a single crystal extracted from a mixture of phases and it was formed in a reaction in which the intended SDA, benzylviologen dichloride, decomposed during the reaction to form 4,4′-dipyridyl, which was found in the 14-ring channels together with pyridine, itself also used in the synthesis.^24^ To our knowledge DIPYR-GaPO has never been produced in a pure form.

**Fig. 6:**
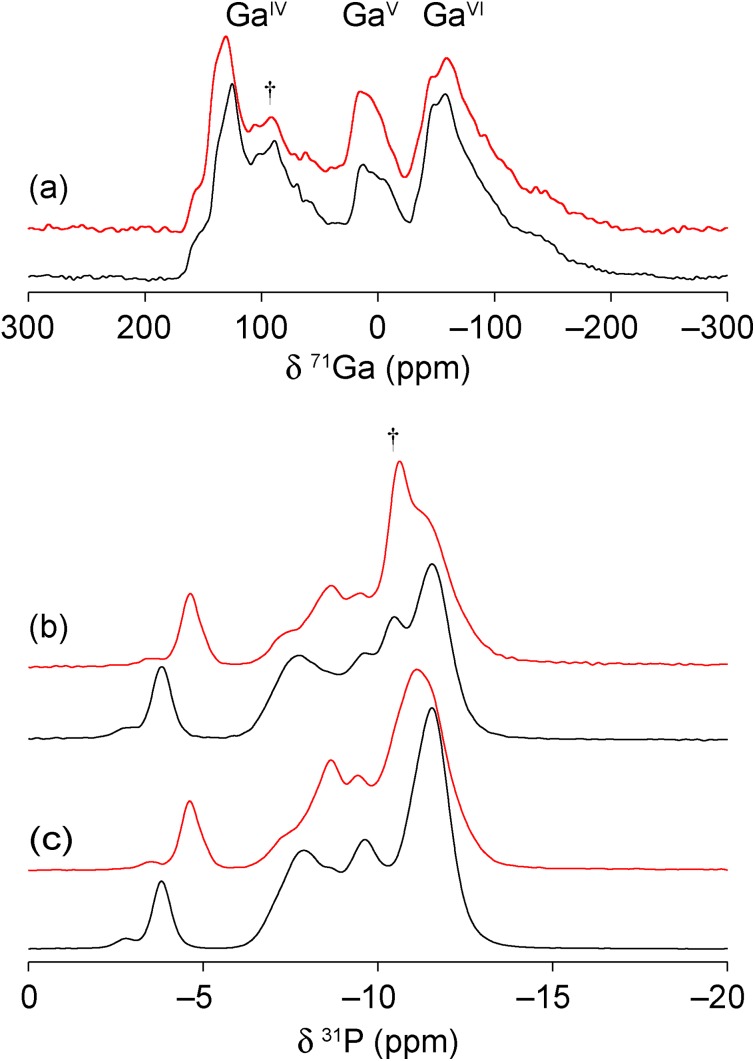
(a) ^71^Ga (20.0 T, 55 kHz) MAS NMR spectra, ^31^P (14.1 T, 14 kHz) (b) MAS and (c) CP MAS NMR spectra of GaPO-34A(mim) (black) and GaPO-34A(pyr) (red). † denotes GaPO_4_ berlinite impurity and the Roman numerals coordination numbers.

The two new gallium phosphates were also studied using solid-state NMR spectroscopy owing to its sensitivity to the local structure, without any reliance on long-range order, making it very complementary to diffraction.^30^ High-field ^71^Ga magic angle spinning (MAS) NMR spectra, shown in Fig. 6(a), show the presence of 4-, 5- and 6-coordinate Ga (Ga^IV^, Ga^V^ and Ga^VI^, respectively) with an (approximate) integrated intensity ratio of 2 : 2 : 3. This is in good agreement with the presence of 7 distinct types of gallium in the crystal structure models, and their predicted coordination environments. However, accurate integration is not possible owing to the extensive overlap of the resonances (even at 20.0 T) and, additionally, the small amount of berlinite present (*δ*_iso_ = 111.1 ppm) will contribute to the intensity of the Ga^IV^ signal. The ^31^P MAS NMR spectra (Fig. 6(b)) show multiple overlapping and broadened resonances in the region of –3 to –14 ppm, characteristic of gallium phosphates (*cf.* 0 to –20 ppm for GaPO-34 27). As for the ^71^Ga NMR spectra, there is a small contribution to the spectrum from the berlinite impurity (*δ*_iso_ = –10.4 ppm),^27^ making it difficult to obtain unambiguously the number and integrated intensity ratios for the P sites. As shown in Fig. 6(c), cross polarisation (CP) from ^1^H was used to filter out the signal from berlinite, which contains no H, allowing 6 major resonances to be defined at –3.8, –7.8, –8.7, –9.8, –11.1 and –11.7 ppm (GaPO-34A(mim)) and –4.4, –7.3, –8.4, –9.3, –11.1 and –11.4 ppm (GaPO-34A(pyr)), although with unequal intensities in both materials. It should be noted that the unequal intensities are also observed for the ^31^P MAS NMR spectra and do not, therefore, arise from the potentially non-quantitative nature of the CP experiment. All ^31^P resonances were equally enhanced by CP, confirming that no P–OH environments are present (as might be expected in an interrupted or partially condensed open-framework phosphate, *e.g.*, cloverite).^31^ For the related aluminophosphate frameworks, ^31^P chemical shifts have been shown to depend on both the mean P–O bond length and P–O–Al bond angle.^32^ As most of the resonances shown in Fig. 6 appear in a narrow chemical shift range, this suggests that there is little variation in the local geometry for most of the P sites. However the most downfield resonances in both materials are suggestive of smaller P–O–Ga angles, which typically occur in small ring structures.^33^ This downfield resonance may, therefore, be tentatively assigned to phosphorus bonded to bridging oxygens that have the most strained bond angles (mean P–O–Ga angle of 132.0° and 129.4° for GaPO-34A(mim) and GaPO-34A(pyr), respectively).

**Fig. 7:**
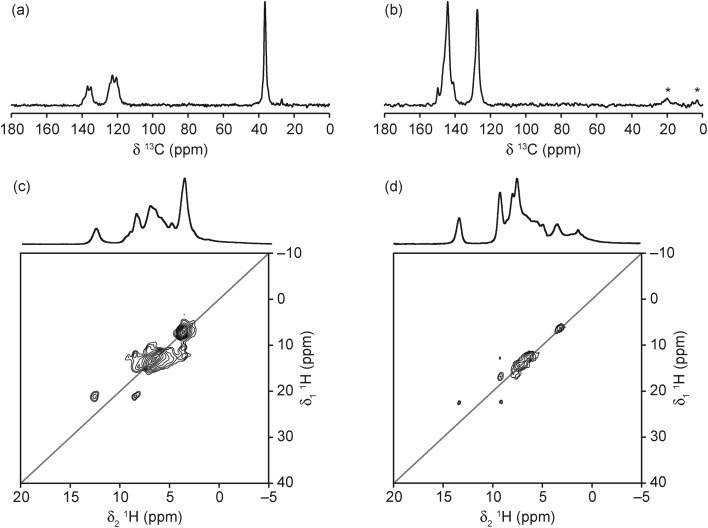
(a, b) ^13^C (14.1 T and 9.4 T, 12.5 kHz) CP MAS NMR spectra, (c, d) ^1^H (14.1 T, 50 kHz) MAS and ^1^H (14.1 T, 60 kHz) DQ MAS NMR spectra (using 2 blocks of BABA pulses for excitation and conversion) of (a, c) GaPO-34A(mim) and (b, d) GaPO-34A(pyr). * denotes spinning sidebands.

The ^13^C CP MAS NMR spectra of the two materials (Fig. 7(a and b)) confirm the presence of the protonated SDA, with resonances at very similar positions to those observed by Amri *et al.* for GaPO-34 materials made with the same SDAs.^27^ However, the complex and broadened lineshapes suggest that, in both materials, the SDAs have multiple orientations, in agreement with the crystal structure obtained for the 1-methylimizadole form, although the number of resonances observed suggest that both SDAs are more disordered than the crystallographic refinement suggests. It should be noted that, in agreement with the carbon content from the refined crystal structures, the signal intensity obtained is comparable to that for GaPO-34,^27^ in contrast to the observation made by Schott-Darie *et al.*^12^ that the ^13^C signal intensity was much lower than for GaPO-34, suggesting that GaPO-34A contained less SDA per formula unit. The ^1^H MAS NMR spectra of the materials (Fig. 7(c and d)) indicate that the SDAs are both protonated, with N**H** resonances observed at 12.6 and 13.3 ppm for mim and pyr, respectively. Full spectral assignment is not possible, however, owing to the extensive overlap of resonances, even at the high MAS rate employed. The ^1^H DQ MAS NMR spectra (also shown in Fig. 7(c and d)) allow further spectral assignment, with the resonance at 3 ppm (in the MAS spectrum of GaPO-34A(pyr)) identified as **H**_2_O owing to the strong on-diagonal correlation. However, this resonance is obscured by the C**H**_3_ resonance in the mim form. The resonances between 4 and 5 ppm in the MAS spectra of both materials are not observed in the DQ MAS spectra (which require spatially close ^1^H pairs), suggesting that these may arise from OH^–^ groups.

**Fig. 8:**
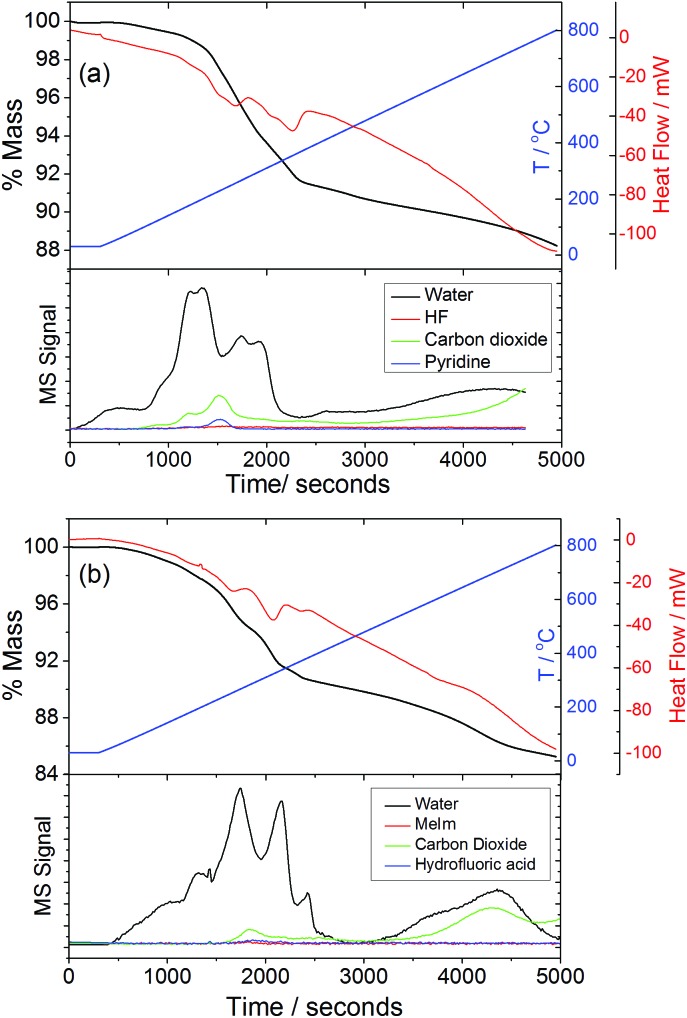
TGA-DSC-MS traces for (a) GaPO-34A(pyr) and (b) GaPO-34A(mim) recorded in air at 10 °C min^–1^.

Thermogravimetric analysis of the two forms of GaPO-34A, Fig. 8, shows onset of mass loss on heating in nitrogen at less than 200 °C and this continues gradually to 900 °C without reaching a plateau. The initial mass loss is accompanied by loss of water seen in the mass spectrum of the evolved gas, which suggests that loss of crystal water and/or dehydroxylation takes place before the combustion of the SDA. Indeed, the evolution of CO_2_ occurs at higher temperature and is observed until the end of the experiment. The total mass loss to 900 °C shows that not all the organic is removed: for GaPO-34A(pyr) a mass loss of 17.4% is expected (observed ∼12%) assuming loss of fluoride, water and all organic to give the neutral “Ga_7_P_6_O_24_(OH)_2_F” and for GaPO-34A(mim) a mass loss of 18.2% is expected (observed ∼14%). The fact that CO_2_ evolution is still observed at 900 °C shows that not all carbon is burnt off. This is consistent with results from related materials, as often reported for gallium phosphates and phosphites mass loss in thermogravimetry is less than expected due to retention of carbon in the material.^34^ Indeed we found in additional TGA experiments (not shown) that prolonged heating above 1000 °C in air was needed to reach a plateau in mass.

**Fig. 9:**
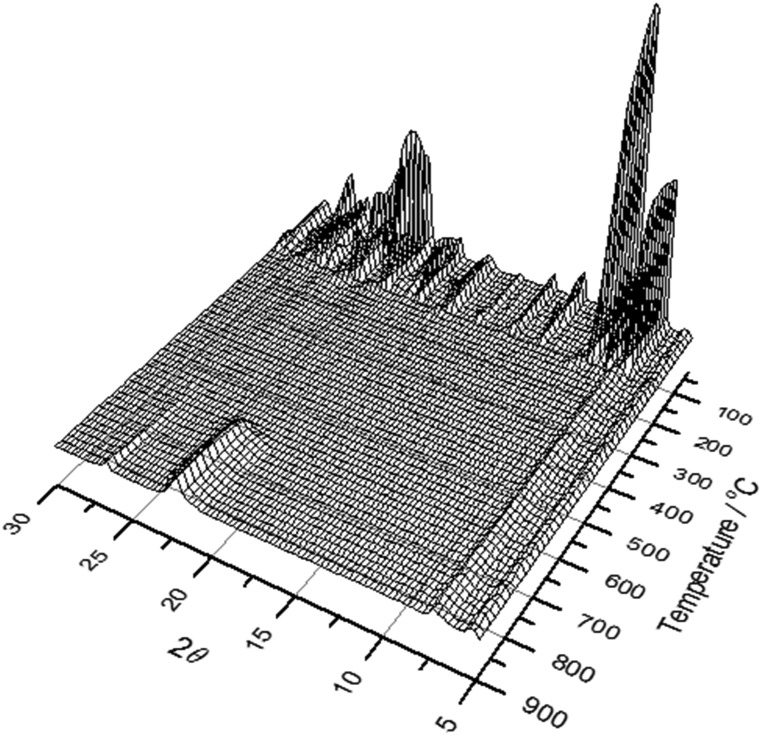
Thermodiffraction measured during heating GaPO-34A(mim) in air from room temperature to 900 °C.

Attempts to remove the SDA from GaPO-34A by calcination to leave an open framework material proved impossible. Thermodiffraction experiments show an abrupt loss of crystallinity on heating to under 150 °C, as seen in Fig. 9. This suggests a complete collapse of the framework and, indeed, no crystallinity is regained until above 600 °C when the low cristobalite polymorph of dense GaPO_4_ is formed (not shown). This behaviour is in stark contrast to GaPO-34, which is stable on heating to above 300 °C, above which the SDA and bridging fluoride can be removed upon further heating to yield the calcined chabazite-like GaPO_4_ with solely tetrahedral gallium, maintaining the open inorganic framework of the as-made material. Thus, although GaPO-34 and GaPO-34A can be prepared with the same pair of SDAs, their thermal behaviour is very different. We also note that there is no transformation of GaPO-34A into GaPO-34 on heating in air (Fig. 9) or under hydrothermal conditions, where collapse of GaPO-34A to dense GaPO_4_ occurs (Fig. S5†). While the collapse of GaPO-34A may be due in part to its lower framework density, and that the Ga : P ratio is not 1 : 1, such that GaPO-34 or a GaPO_4_ polymorph cannot be formed directly, we also note that the variety of bridging anions in the structure, compared to the single bridging fluoride in GaPO-34, is likely to lead to a destabilised inorganic framework upon their loss, with Ga coordination environments and bond angles unable to support a truly porous framework. Indeed, Girard *et al.* found using computational simulation that many GaPO structures are energetically unstable with respect to berlinite upon SDA removal^7^ and GaPO-34A would appear to fall into this category. ^13^C CP MAS NMR analysis of the final product of the *in situ* XRD experiment shows that, despite the loss of crystallinity and the collapse of the open framework, the solid product contains carbon species that resemble imizadole-containing organic fragments (ESI†). This is consistent with the TGA analysis that show that removal of all organic requires prolonged heating at high temperatures (see above).

## Conclusions

We have provided the first example in the chemistry of open-framework phosphates of how the choice of reagents (gallium source) and aging of the gel precursor can dictate which crystalline phase is formed on subsequent identical thermal treatment of the gel. In this case we have isolated and determined structures of two new variants of a material previously only found in a multi-phase mixture. Such gel aging effects have been long known in the case of silicate zeolites, and, although still not fully understood, it has been postulated that the structural building units for the formation of microporous structures are formed under ambient conditions in the gel and can evolve with time, leading to different products dependent on the history of the gel before it is heated. Unlike the formation of zeolites under basic conditions, the formation of phosphate occurs at low pH, often from less viscous reaction mixtures, but it is now apparent that similar aging effects can take place. This leads to the observation that in the discovery of new porous solids understanding the gel chemistry, with its inherent structural disorder, must remain an important target in materials chemistry: this requires non-standard experimental methods such as solid-state NMR in custom-designed heated rotors, or high-energy synchrotron X-ray diffraction experiments to follow phase evolution in realistic reaction cells, and will be the subject of future work. It may be the case that similar gel aging effects occur in other gallium phosphate syntheses, which in turn may lead to the discovery of new phases, and this must also be the topic of further investigation.

## Conflicts of interest

There are no conflicts to declare.

## Supplementary Material

Supplementary information

Crystal structure data
