# Supplementary material for: A gel aging effect in the synthesis of open-framework gallium phosphates: structure solution and solid-state NMR of a large-pore, open-framework material
Source: Dalton Trans. 2017 Nov 16;46(48):16895–904. doi: 10.1039/c7dt03709k (PMC5789431; doi:10.1039/c7dt03709k)
Supplement: Supplementary file 1 [file DT-046-C7DT03709K-s001.pdf]

Electronic Supplementary Material (ESI) for Dalton Transactions.
This journal is © The Royal Society of Chemistry 2017

SUPPORTING INFORMATION FOR:

A Gel Aging Effect in the Synthesis of Open-Framework Gallium Phosphates: Structure Solution and Solid-State NMR of a Large-Pore, Open-Framework Material

Lucy K. Broom, Guy J. Clarkson, Nathalie Guillou, Joseph E. Hooper, Daniel M. Dawson, Chiu C. Tang, Sharon E. Ashbrook and Richard I. Walton*

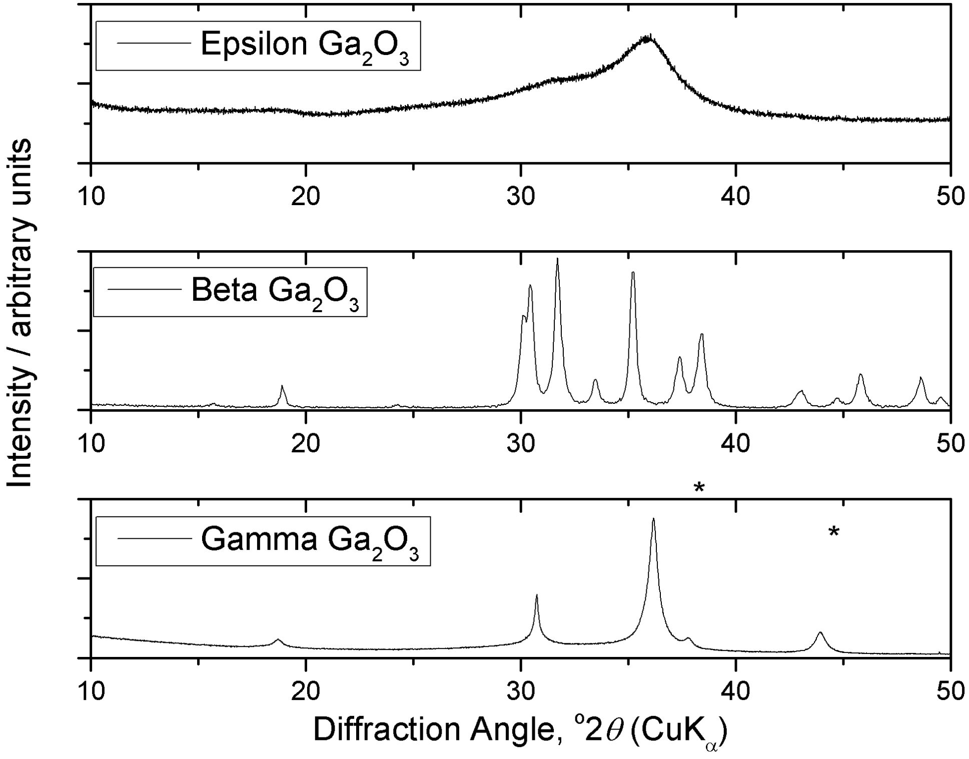

Figure S1: PXRD patterns of the gallium oxide reagents used in synthesis. For γ-Ga_2O_3, the * indicate peaks due to the sample holder.

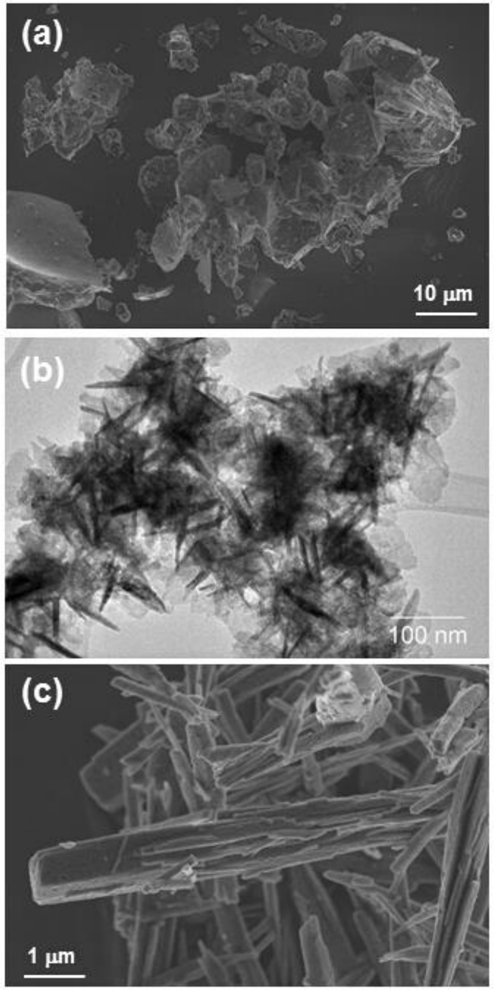

Figure S2: Electron micrographs measured from Ga_2O_3 precursors showing their different particle morphologies: (a) ε-Ga_2O_3 (b) γ-Ga_2O_3 (c) β-Ga_2O_3. (a) and (c) were recorded using a Zeiss Supra 55-VP Field Emission SEM and (b) with a JEOL 2100 TEM-STEM.

Table S1: Results of exploratory synthesis of gallium phosphates from gels of composition $1Ga_2O_3$:$2H_3PO_4$:1HF:nH_2O:1.7mim. After the aging period all gels were heated under hydrothermal conditions for 24 hours.

**Ga_2O_3 Source**	**Water equivalents (*n*)**	**Ageing time / h**	**GaPO Product from PXRD**
Poorly crystalline ε-Ga_2O_3	70	0	GaPO-34A
	70	1	GaPO-34A
	70	2	GaPO-34
	70	3	GaPO-34
β-Ga_2O_3	70	1	GaPO-34A

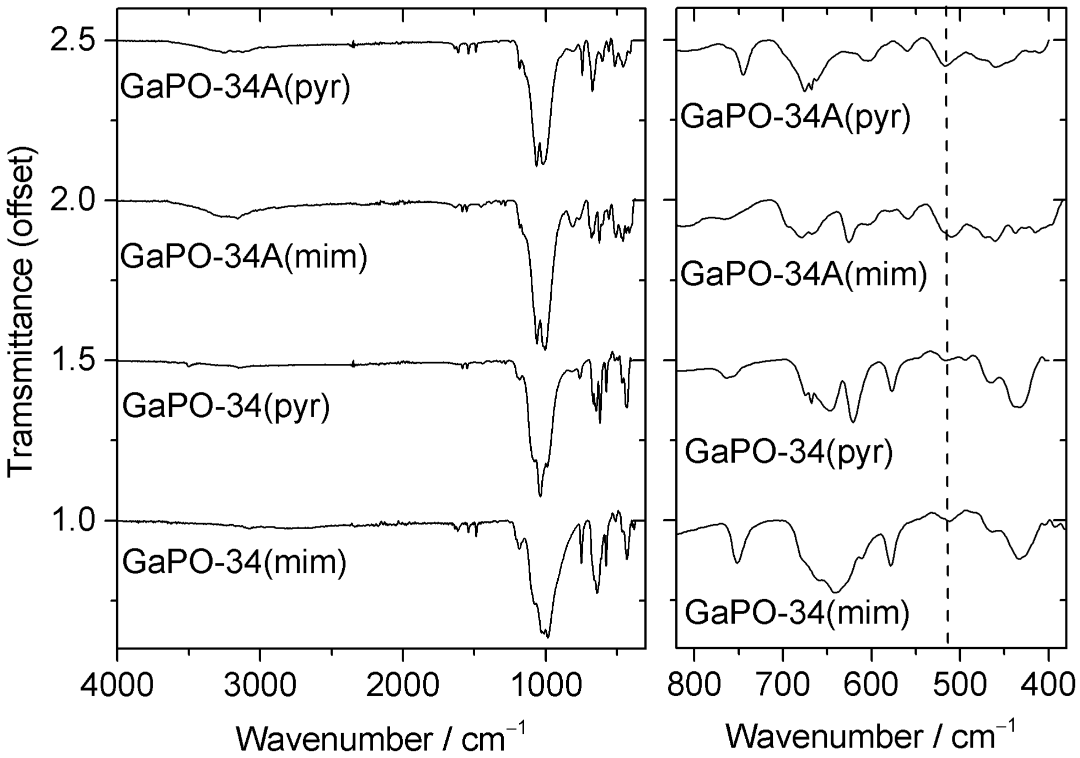

Figure S3: IR spectra of GaPO-34A(mim) and GaPO-34A(pyr) compared to GaPO-34(mim) and GaPO-34(pyr). The expansion in the right panel is the low wavenumber region with the dotted line showing the position of the Ga-F stretch.

Table S2: Bond distances and bond valences for GaPO-34A(pyr)

		Distance / Å	Bond Valence (**Total**)			Distance / Å	Bond Valence (**Total**)			Distance / Å	Bond Valence (**Total**)			Distance / Å	Bond Valence (**Total**)
Ga1	O13	1.844	0.74	Ga2	O61	1.794	0.84	Ga3	F3	1.878	0.50	Ga4	O21	1.865	0.70
	O64	1.848	0.73		O1	1.859	0.71		O42	1.882	0.66		F3	1.867	0.51
	O51	1.878	0.67		O32	1.862	0.70		O11	1.909	0.62		O14	1.939	0.57
	O52	1.905	0.62		O22	1.904	0.62		O41	1.925	0.59		O1	1.948	0.55
	F1	2.193	0.21						O12	1.953	0.55		O24	1.949	0.55
							**2.87**		F2	1.980	0.38		O3	1.959	0.54
			**2.97**												
											**3.29**				**3.42**
Ga5	O23	1.762	0.92	Ga6	O54	1.746	0.96	Ga7	O44	1.899	0.63				
	O53	1.799	0.83		O63	1.785	0.86		O34	1.916	0.61				
	O33	1.800	0.83		O43	1.815	0.80		O4	1.918	0.60				
	O4	1.885	0.66		O62	1.839	0.75		O2	1.930	0.58				
	F1	2.231	0.19						F2	1.966	0.39				
							**3.36**		O31	1.975	0.38				
			**3.42**												
											**3.20**				
P1	O13	1.549	1.16	P2	O21	1.532	1.21	P3	O33	1.521	1.25	P4	O44	1.517	1.26
	O11	1.560	1.13		O24	1.538	1.20		O31	1.525	1.24		O41	1.531	1.22
	O12	1.574	1.08		O23	1.547	1.17		O34	1.535	1.21		O42	1.542	1.18
	O14	1.589	1.04		O22	1.555	1.14		O32	1.553	1.15		O43	1.571	1.09
			**4.41**				**4.72**				**4.84**				**4.76**
P5	O53	1.532	1.21	P6	O64	1.494	1.35								
	O54	1.552	1.15		O62	1.552	1.15								
	O51	1.555	1.14		O61	1.553	1.15								
	O52	1.557	1.14		O63	1.564	1.11								
			**4.64**				**4.76**								

Table S3: Bond distances and bond valences for GaPO-34A(mim)

		Distance / Å	Bond Valence (**Total**)			Distance / Å	Bond Valence (**Total**)			Distance / Å	Bond Valence (**Total**)			Distance / Å	Bond Valence (**Total**)
Ga4	F1	1.926	0.44	Ga1	O2	1.903	0.63	Ga2	O5	1.799	0.83	Ga3	O9	1.707	1.07
	O15	1.935	0.58		F1	1.915	0.45		O8	1.807	0.81		O12	1.743	0.97
	O14	1.962	0.53		O13	1.937	0.57		O6	1.838	0.75		O11	1.872	0.68
	F1	1.926	0.44		O4	1.953	0.55		O3	1.843	0.74		O10	1.930	0.58
	O15	1.935	0.58		O6	1.973	0.52		F3A	2.114	0.26		F3A	2.521	0.09
	O14	1.962	0.53		O1	2.063	0.41								
			**3.09**				**3.12**				**3.39**				**3.38**
Ga3A'	O11	1.620	1.35	Ga3'	O9	1.707	1.07	Ga2'	O5	1.799	0.83				
	O10	1.703	1.08		O12	1.743	0.97		O8	1.807	0.81				
	O12	1.937	0.57		O11	1.872	0.68		O6	1.838	0.75				
	O9	2.240	0.25		O10	1.930	0.58		O3	1.843	0.74				
			**3.25**				**3.29**				**3.13**				
P3	O13	1.521	1.25	P1	O2	1.509	1.29	P2	O9	1.518	1.26				
	O14	1.524	1.24		O3	1.525	1.24		O8	1.524	1.24				
	O15	1.526	1.23		O4	1.528	1.23		O11	1.534	1.21				
	O12	1.548	1.16		O5	1.536	1.20		O10	1.536	1.20				
			**4.89**				**4.96**				**4.91**				

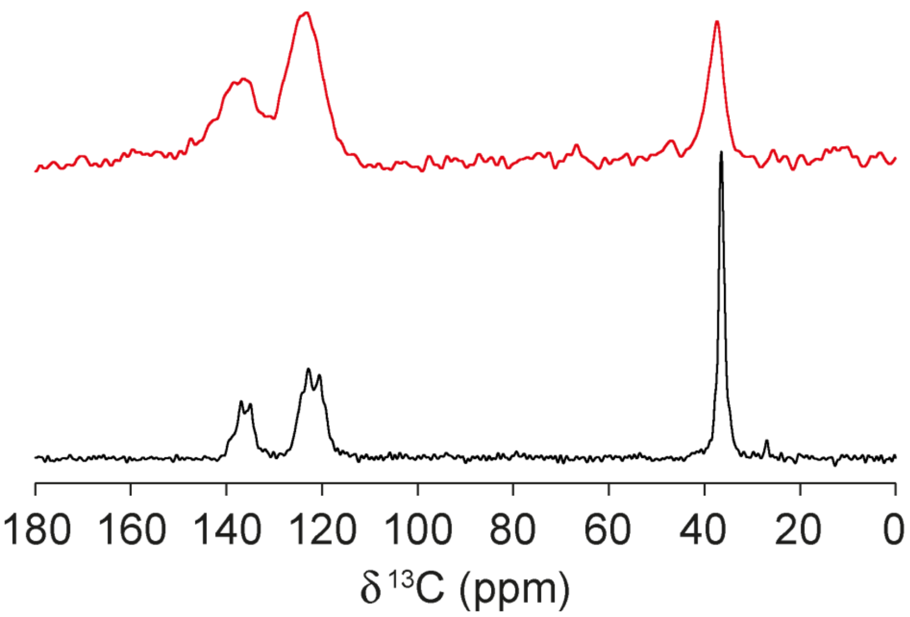

Figure S4: ^{13}C (9.4 T, 12.5 kHz) CP MAS NMR of the collapsed GaPO-34A(mim) following the *in situ* XRD experiment (red) compared to the spectrum of GaPO-34A(mim) (black) prior to calcination.

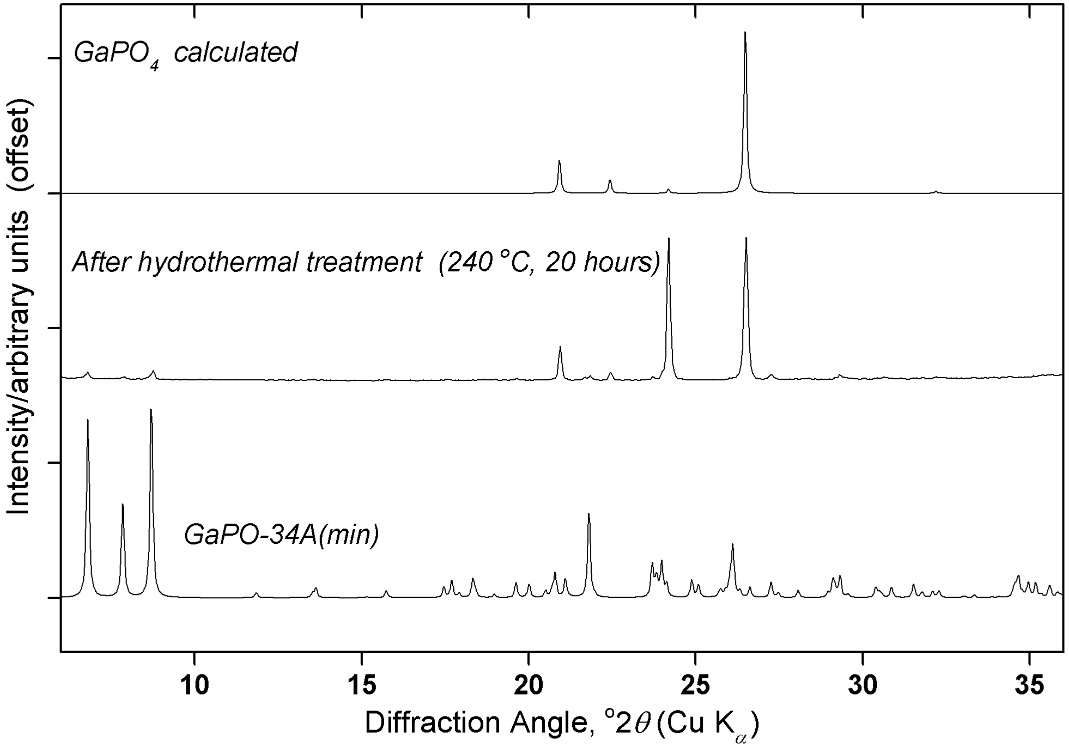

Figure S5: Powder XRD of GaPO-34(mim) after hydrothermal treatment showing collapse into dense $GaPO_4$ (quartz-type). The $GaPO_4$ pattern was calculated from the crystal structure published in Goiffon *et al. Rev. Chim. Miner.* (1983) 20**, 338-350.**
